# Functionality and Applicability of Starch-Based Films: An Eco-Friendly Approach

**DOI:** 10.3390/foods10092181

**Published:** 2021-09-14

**Authors:** Sneh Punia Bangar, Sukhvinder Singh Purewal, Monica Trif, Sajid Maqsood, Manoj Kumar, Vishal Manjunatha, Alexandru Vasile Rusu

**Affiliations:** 1 Department of Food, Nutrition and Packaging Sciences, Clemson University, Clemson, SC 29634, USA; vishal.93.manjunath@gmail.com; 2 Department of Food Science & Technology, Maharaja Ranjit Singh Punjab Technical University, Bathinda 151001, India; purewal.0029@gmail.com; 3 Centre for Innovative Process Engineering (CENTIV) GmbH, Food Research Department, 28816 Stuhr, Germany; monica_trif@hotmail.com; 4 Department of Food, Nutrition and Health, College of Food and Agriculture, United Arab Emirates University, Al Ain 15551, United Arab Emirates; sajid.m@uaeu.ac.ae; 5 ICAR—Central Institute for Research on Cotton Technology, Mumbai 400019, India; manoj.kumar13@icar.gov.in; 6 Department of Food Science, Life Science Institute, University of Agricultural Sciences and Veterinary Medicine Cluj-Napoca, 400372 Cluj-Napoca, Romania

**Keywords:** starch, films, properties, mechanical, barrier, applications

## Abstract

The accumulation of high amounts of petro-based plastics is a growing environmental devastation issue, leading to the urgent need to innovate eco-safe packaging materials at an equivalent cost to save the environment. Among different substitutes, starch-based types and their blends with biopolymers are considered an innovative and smart material alternative for petrol-based polymers because of their abundance, low cost, biodegradability, high biocompatibility, and better-quality film-forming and improved mechanical characteristics. Furthermore, starch is a valuable, sustainable food packaging material. The rising and growing importance of designing starch-based films from various sources for sustainable food packaging purposes is ongoing research. Research on “starch food packaging” is still at the beginning, based on the few studies published in the last decade in Web of Science. Additionally, the functionality of starch-based biodegradable substances is technically a challenge. It can be improved by starch modification, blending starch with other biopolymers or additives, and using novel preparation techniques. Starch-based films have been applied to packaging various foods, such as fruits and vegetables, bakery goods, and meat, indicating good prospects for commercial utilization. The current review will give a critical snapshot of starch-based films’ properties and potential applicability in the sustainable smart (active and intelligent) new packaging concepts and discuss new challenges and opportunities for starch bio composites.

## 1. Introduction

Packaging of eatable materials is considered a broad-spectrum tool that indicates its nutrients detail, manufacturing, and expiry information, making it convenient for customers. Food products are available in many forms, such as semi-solid, solid, and liquid phases, making it necessary to pack them accordingly. Efficient packaging enables protection against dust, undesirable pollutants, and protects the food material from microbial contamination [1]. To make the food products more demandable in the market, they should be packed in material with enough strength to hold and keep quality and safety to ensure the prolonged shelf life of goods. Since industrial development, different packing materials have been explored to provide the safety features of food materials. Initially, metal was preferred for packing as it has excellent malleability features and lightweight and heat resistant.

Further, paper packing is recommended for a wide range of applications; however, they have a lower moisture barrier with non-significant mechanic strength. After that, plastic was used as an alternate material for packing purposes as it has both mechanical strength and moisture barrier. However, their long-term environmental persistence creates pollution problems and an unnecessary burden on food processing industries. Therefore, it raises the need for an alternate packing material that is safe for consumption and is biodegradable.

Starch, a biologically important macromolecule, is considered the most promising polymer of natural origin as it has overwhelming biodegradability, significant abundance, and cost-effective nature [2,3]. Starch’s easy availability and annual renewable nature make it the perfect base material for broad-spectrum industrial applications. Starch has diverse applications in the food industry, particularly as a thickening agent (e.g., to modify texture, viscosity) and for water retention (e.g., swelling properties); therefore, there is an increasing demand for starch. Maize is the main source of starch isolation (wet and dry milling), followed by potato, wheat, rice, and cassava. Cereal starches (maize, rice, and wheat) are used as ingredients or influencing ingredients in foods in both native and modified conditions.

Modified starches, based on physicochemical characteristics and modifying agents, and on their production, are classified into the following categories:

(i) Pregelatinized: physically modified by extrusion or drum drying;
(ii) Derivatized: chemically modified with modifying agents such as acetyl, hydroxypropyl, phosphate;
(iii) Crosslinked: chemically modified using epichlorohydrin, trimetaphosphate;
(iv) Dextrinized: physically modified by irradiation, heat (pyrodextrinization); chemically modified: oxidizing agents, acid hydrolysis; enzymatically modified: amylolytic enzymes.

By combination, chemically modified derivatization and crosslinking obtain double-derived starches. Starch in food products imparts physicochemical and functional characteristics (composition, crystallinity, gelatinization behavior) [4].

Edible films and coating are attracting attention at an industrial scale as they have enough potential to combat rising pollution problems. Starch has remarkable properties, including biodegradability, sustainability, abundance, and can be modified or blended with other polymers. Various starches have been investigated for food packaging applications, including cereals, millets, and pulses starches. Starch could be used as base blending material to prepare eco-friendly packing material [5,6]. Adequate packing uses two different approaches (1) use of edible source as a coating material for fruits/vegetable surfaces, (2) a materialistic approach which could be further divided as (a) gel conversion (biopolymer), (b) thin edible films from gels [7].

A thin layer of the edible film may be applied to protect the food material from dust and preserve them in a fresh stage. To decrease the waste production rate, biodegradable polymers may be used [8].

Besides their biodegradability, biopolymers have other important properties/features, such as low temperature, seal ability, air permeability, availability, and low cost. Biopolymers, especially different types of starch, chitosan, whey, gums (seaweed-based gums (e.g., alginate, carrageenan)), plant-origin gums (e.g., mastic gum), and microbial gums (e.g., xanthan gum, cellulose), are recommended for coatings and packaging purposes alone or in blends to improve the mechanical properties [9,10,11,12].

Biopolymers, as model systems, have an incredible ability to undergo gelation upon the addition of multivalent cations, leading to the crosslinking and aggregation of both when in contact with cations. Due to the exchange of divalent ions during the reticulation process, the creation of a strong network via gelation occurs, which, in turn, leads to a stronger matrix (in terms of structural and mechanical properties) [13,14,15,16].

The ionic crosslinking of biopolymers can successfully produce aerogels, typically produced by the sol–gel technique. The aerogels are used for advanced intelligent food packaging materials applications. Starch suspensions are initially crosslinked to increase aerogel hardness (and at this point, if needed, loaded with antioxidant, antimicrobial, or other active ingredients), followed by gelling and then freeze-dried to corresponding aerogels [17,18,19].

The investigation of biopolymer interactions in gels is ongoing research. It has become increasingly critical for a better understanding of the behavior of biopolymer blends which is necessary for further applications. Due to these rapid form gels of the biopolymers, their interest and applications have increased in recent years in specific low-cost three-dimensional (3-D) shapes and patterns printing (3D printing) [20,21,22].

The film and coating layer features of starch enable its use for commercial purposes [23]. Rising environmental issues due to plastic as a packaging material are the drivers for preparing an informative review on biodegradable material of plant origin.

A recent study published this year has shown that the research on “starch food packaging” is still beginning, based on the low number of studies published in the last decade in Web of Science [24]. Therefore, starch-based coatings and packaging still represent a challenging issue—how to make it an intelligent and active, actually a smart packaging material [25,26,27,28]. This review paper provides in-depth information on the preparation, properties, and applications of starch-based films.

## 2. Technique to Prepare Starch-Based Films

The capability of film formation is a fascinating feature of starch. Starch is not soluble in cold water and does not melt like conventional plastics since the starch degradation temperature is less than the melting point. Nevertheless, starch granules lose their semi-crystalline behavior irreversibly and are transformed into a continuous matrix when starch is subjected to force, heat, and plasticizers [29]. This characteristic of starch is used to formulate biodegradable films. The transformation of starch into films is described by solution casting or extrusion processing as the most commonly used techniques.

### 2.1. Solution Casting

Solution casting is one of the commonly used techniques to prepare starch films. Solution casting involves (a) solubility of the biopolymer in a solvent/plasticizer, (b) casting in the mold and, (c) drying. The process requires starch gelatinization by mixing starch in water (3–12%) followed by heating above T_gel_ [30]. The swelling behavior of water exclusively describes the gelatinization process due to which amylose and amylopectin chains break, which makes these chains fully solubilized. Gelatinized and a solubilized solution is further cast into a mold followed by a drying process to get polymer film after evaporation of the solvent. The drying step is crucial to improve intramolecular interaction between the amylose and amylopectin chains [31]. Drying equipment, including microwaves, hot-air ovens, trays, and vacuum driers, is used for the casting process for easy evaporation of solvents and peeling of films [32]. Quick-drying methods for the casting process have exhibited negative physical and structural behavior [33].

Water has been proven to be an idol plasticizer/solvent for starch; however, as it evaporates, films become brittle. Therefore, it is suggested to mix water with other plasticizers, including glycerol, sorbitol, simple sugars, and urea, to access conditions fit for solution casting. Plasticizers are essential for films since they enhance the flexibility of films by decreasing intermolecular H-bonding because it enhances the intermolecular space. In addition, they improve the mechanical characteristics of starch-based films for packaging applications. This process is considered an energy and time-consuming method since water is the only cost-effective solvent for starch. Since this method is not a commercial-scale technique for film formulations, it displays an advantage over all other film-forming methods; the films prepared by the casting method exhibit an excellent aesthetic quality, an essential feature for food packaging materials.

The nonionic, cationic, and anionic starch-based films obtained using the casting method activated with the cationic surfactant lauroyl alginate possess antimicrobial activity. They are ideal candidates to extend the shelf-life of the packaged food products [34]. Cassava starch films incorporated with propolis extract and cellulose nanocrystals were found to inhibit the proliferation of *Staphylococci* in sliced cheese during 28 days of storage. However, without propolis, ethanolic extract and commercial PE film show no antimicrobial activity [35]. Further, potato starch-based biodegradable and antimicrobial nanocomposite films were prepared with a constant concentration of zinc oxide nanoparticles using the casting method. Authors reported that the films prepared with clove oil were most effective against *S. aureus* (22–100% inhibition), those prepared with cinnamon oil were effective against *C. jejuni* (19–22% inhibition), and growth of *E. coli* was inhibited (33–40% inhibition) to the maximum extent by potassium sorbate incorporated films [36].

Several intelligent starch-based films blends with PVA, and nanoparticles have been developed for simultaneous colorimetric indication or pH indication and at the same time with antimicrobial activity for food packaging applications. Changes in the chemical environment of food are very important for product safety, and incorporating such indicators is of great interest [37,38].

Significant mechanical and barrier enhancement have been reported by different studies using the casting method to obtain poly(lactic acid)/nanoclay composite film or starch/nanoclay composites prepared by employing organically modified montmorillonite (MMT) minerals (poor dispersion and presence of large agglomerates) [39,40].

### 2.2. Extrusion Process

The extrusion process is another widely used and generally preferred processing method to produce polymer films and is a commercially used polymer processing method. This technique alters structural properties and enhances the physiochemical characteristics of extruded substances [41]. The extrusion process is divided into three zones: (a) the feeding, (b) the kneading, and (c) the heating zone [42]. Polyethylene glycol or sorbitol (10–60%) are commonly used as plasticizers for the extrusion process [43].

Mechanical and thermal energy are the two key factors involved in the extrusion technique to formulate extruder-based film [41]. Screw speed also has some impact on specific mechanical energy [44]. Different screw speed affects film properties, i.e., homogeneity, shear rate, and stress, and control the residence time, providing opportunities to add and remove additives, including stabilizers. As screw speed is increased, the torque value of the extrusion process to obtain films is decreased [42]. Changes in screw speed significantly modulate the specific properties of edible films as it affects the shear stress, shear rate, and homogeneity while controlling residence time.

Further, the variation in screw speed allows the removal or addition of suitable additives (stabilizers). The decrease in torque value of edible films concerning screw speed depends on the thermoplastic behavior of polymers during heating and plasticization.

This process depends on polymers’ thermoplastic behavior whenever plasticization and heating occur above the glass transition temperature (T_g_) and reduced water level conditions [45].

Other parameters, including feed moisture content, screw speed, barrel temperature, die diameter, pressure at the die, energy input, etc., are essential for the extrusion process to influence the final products. The co-extrusion method could be used to prepare a multilayer with improved flexibility. The multilayer enhances the functionality, processability, and design of the multilayer film structure [46]. Compared with the casting process, this technique has a short processing time with low energy consumption with improved mechanical (elongation at break) and optical properties (transparency), respectively [47]. Furthermore, extrusion processing is a high-performance, low-cost, and efficient commercially used method in the food sector. However, the extrusion process limits temperature tolerance and low moisture raw material blends, restricting specific polymers.

Flores et al. [48] prepared edible films by extrusion technology from tapioca starch by adding xanthan gum and potassium sorbate. The results reported that potassium sorbate reduced the tensile strength and young modulus and improved strain at break. Moreover, xanthan gum reinforced the films with enhanced water solubility and decreased moisture content. Xanthan gum added to cassava starch improved the tensile behavior, stress, and strain at break properties of the starch film obtained by casting and extrusion methods [15,49].

Nano-ZnO and nano-SiO_2_ nanoparticles can be used as composite reinforcing agents to prepare starch-based films through extrusion blowing [50]. Nano-ZnO and nano-SiO_2_ could also improve the surface smoothness of the film. González-Seligra et al. [51] determined conditions of the extrusion process for food packaging on their morphology and functionality. The authors evaluated the effect of screw speed on extruded thermoplastic starch materials. The process at 80 rpm had the highest strain at break, and at 40 and 120 rpm reported to have the best modulus and stress at break. The authors observed the processing at 120 rpm as the best option for thermoplastic films due to greater modulus, tensile strength, and slower starch retrogradation.

For thermoplastic starch, a film can be obtained with improved mechanical properties and water vapor permeability when blended with nanoclays by extrusion and thermopressing, strongly associated with the dispersion of nanoclay in the polymer matrix [52,53,54].

## 3. Properties of Starch-Based Films

### 3.1. Barrier Properties

Barrier properties are considered one of the important determinant factors as these properties decide the shelf life of packed food material. Barrier properties help to maintain the moisture level in packed food as well as helps in eradicating microbial contaminations from surrounding environmental conditions [55,56]. Hiemenz and Rajagopalan [57] observed that films (edible) and coatings prepared using proteins and carbohydrates pose significantly fewer barriers towards moisture. The fewer moisture barrier properties in these films and coating may be due to the hydrophilic nature. Further, to enhance the moisture barrier level, some hydrophobic compounds are used, especially lipids. A number of studies also advise using surfactants as their use helps reduce surface tension while enhancing moisture barrier and adhesion properties of films [58,59]. Andreuccetti et al. [60] demonstrated that gelatin-based plasticizers (hydrophobic) significantly improved the moisture barrier properties of films. Barrier properties of starch-based films are presented in Table 1.

Water vapor permeability: Water and oxygen are the two important barrier characteristics of biodegradable packaging polymer. These barrier properties directly prevent moisture and oxygen exchange between the product and the surroundings. Delassus [61] investigated the water vapor permeability of mung bean starch (MBS) and observed that films prepared using starch (mung bean) possess non-significant resistance towards moisture barriers. The sole reason behind the poor resistance for moisture is the hydrophilic nature of films (MBS). The value of films for water vapor pressure (WVP) has been found in the range from 0.20 to 0.46 mg·mm/Pa·hr·m^2^. WVP values of films (MBS) was significantly higher as compared to WVP values of plastics (high density polyethylene) (0.01 mg·mm/Pa·hr·m^2^) followed by low density polyethylene (0.03 mg·mm/Pa·hr·m^2^) and (polyethylene terephthalate) (0.05 mg·mm/Pa·hr·m^2^), respectively.

Majzoobi et al. [62] observed that those films prepared using corn starch possess a WVP value of 0.0019 g·mm/m^2^·h·Pa, however, when EF was added in corn starch, the WVP values rose significantly. Further, films prepared using pure EF showed the WVP value as 0.0027 g·mm/m^2^·h·Pa. Higher WVP values might be due to the porous structure of films and irregular surfaces, indicating lipids and protein, and starch in the matrix. Higher WVP values in any films limit their uses at a commercial scale as it may lead to deterioration of the quality of food products packed in them [63]. Ultimately, the WVP value is a determinant factor that could indicate products’ shelf life during storage [64]. Studies on films open a new era for packaging food products as their action determines the life of perishable products during storage conditions. Sharma et al. [65] investigated the WVP values of faba bean starch films and concluded that WVP values vary as 1.10 and 1.34 g·m/Pa·s·m^2^. The authors reported that crosslinking of faba bean starch at different levels (1, 3, and 5%) using sodium trimetaphosphate brings desirable changes in films as confirmed by lower WVP values (modified starch). WVP was observed as native (faba bean) (1.10 g·m/Pa·s·m^2^) and modified starch films (1.34 g·m/Pa·s·m^2^). Further, the level of modification is directly proportional to the changes in WVP values up to certain specific limits depending on the type of botanical starch used. Starch modifications result in improved mechanical strength of films and WVP value [66].

A study on films prepared with native rice starches did not evaluate for WVP because they showed cracks along with the film. The intensity of acetylation significantly influenced the water vapor permeability of high amylose starch films, once the acetylated starch films with 0.42 and 0.72 of substitution degree had a lower WVP than the acetylated starch films with DS = 0.24 [67]. Further, Zamudio-Flores et al. (2007) [68] performed a dual modification of banana starch. The authors oxidized the banana starch at three different levels and acetylated it. They reported that WVP increased with oxidation level, but the acetylation decreased this parameter.

Starch nanocomposites incorporating montmorillonite and crosslinked (e.g., citric acid) were found to reduce the moisture sorption and swell at high relative humidity, improving the barrier properties. Several studies have proved the crosslinking of starch with citric acid to produce a water-insensitive barrier applied for coatings. It is commonly used to produce hydrophilic films of carboxymethyl starch (CMS) and carboxymethyl cellulose (CMC) with improved physicochemical properties [69,70,71,72].

It seems that the addition of CMC to different starch-based films (rice, sorghum, corn, and cassava), which were glycerol-plasticized enhanced water resistance as increased maximum tensile strength [73,74,75,76].

Nanocomposite films obtained by extrusion from different combinations of cassava starch, xanthan gum, and nanoclays (sodium montmorillonite—MMT-Na) were more transparent and resistant, had lower water sorption capacities and lower water vapor permeability, good combination of mechanical properties. The addition of xanthan gum has improved the elongation of starch films [77].

Oxygen vapor permeability: Oxygen vapor permeability (OVP) of starch-based films is well documented. Rompothi et al. [78] prepared starch (mung bean) based films and studied them in their OVP. They observed that films prepared using mung beans have a lower OVP value than commonly used plastic material. Further, many documentations of films suggest the potential of starch (mung bean) toward low OVP, which is capable enough as commercially used plastic polymers [61,79,80]. The OVP value of starch (mung bean) films was 0.23–1.15 cc·μm/m^2^·day·kPa. Inclusion of glycerol (20–30%) while making starch-based films results in a slight decrease in OVP value. Further, compared to other non-plasticized materials, when sorbitol (30–40%) was used, it resulted in a comparatively low OVP value [81]. Thirathumthavorn et al. [82] promulgated that the mung bean starch films that were prepared using sorbitol as plasticized material possess significantly lower OVP value as compared to native/rice starch (acid-treated)/tapioca/rice (acid-treated) reported in previously published data [83,84]. The complexity of starch also affects the different parameters of films in a notable manner [85]. Low OVP value is indirectly proportional to the WVP, as, on this concept, different films are being prepared and further recommended for a variety of products [86]. The parameter that affects the OVP and WVP is the hydrophobic and hydrophilic nature of the material used.

Basiak et al. [87] prepared composite edible films by laminations of wheat starch solution, rapeseed oil, and wheat starch solution as a 3-layer process. They observed that adding lipids in the starch matrix also significantly reduces the OVP by seven times. It was observed that the addition of a lipid component would result in a significant increase in the film water-barrier properties (reduce moisture sorption) and affect the mechanical and oxygen barrier properties. On the contrary, in the case of film-forming emulsions obtained by casting method, the incorporation of the essential oil (cinnamon or ginger) in the starch–sodium caseinate films provoked a slighter increase in the oxygen permeability values due to the rise in oxygen solubility in the oil, and slightly reduced water vapor permeability [88,89,90]. This opposite behavior could be attributed to the lower content of plasticizer used; therefore, where fewer OVP values are required, glycerol could be recommended for film preparation.

Incorporating other kinds of oils (e.g., rapeseed oil, olive oil) seems to have the same positive effect in reducing oxygen and reducing the hygroscopic of the starch-based films [87,91].

The addition of oil reduces the oxygen permeability because the hydrophobic characteristics of the components reduce the water content of the starch-based films and thus their oxygen solubility [92].

### 3.2. Mechanical Properties

The mechanical characteristics, including tensile strength (TS), elongation at breakage (EAB), Young’s and storage modulus, and loss factor (tan δ), are crucial in packaging materials. These mechanical attributes help predict their mechanical potential and evaluate the feasibility of their application as food packaging materials [93].

TS and EAB are primary parameters to characterize film for packaging. TS and EAB demonstrate the potential of food packaging resistance to breakage and maintaining integrity under stress during processing, handling, and storage [94]. The mechanical potential of starch-based films is evaluated using universal testing machines, dynamic mechanical analyzers, or texture meters. Using a universal mechanical testing machine, it could be proved that the addition of nanocellulose exhibited an improved tensile strength of starch-based films. In contrast, incorporating starch nanoparticles decreased the elongation at break [95].

Further, DMA analysis is used to demonstrate dynamic mechanical properties of starch-based films through the changes of storage modulus (E′, stiffness) and loss factor (tan δ, glass transition temperature). Mechanical parameters that determine the commercial status of starch-based films are their tensile strength followed by thickness of the film, moisture level, WVP, elongation at break, and solubility, respectively. The variation in the above-said properties determines the use of films for different purposes. These factors help the researchers to provide relevant information regarding the use of starch films in the market as per the demand of consumers. Mechanical properties of starch-based films are presented in Table 2.

Cassava starch, mung bean starch, and a blend of cassava and mung bean starch with two types of plasticizers (glycerol or sorbitol) were used by Vu et al. [96] for film formation. TS and EAB of plasticized starch films varied from 2.86 to 20.64 MPa and 10.84 to 21.37%. Regardless of plasticizer type, the low amylose content cassava starch films (6.45 MPa) exhibited lower TS than the mung bean starch films (14.99 MPa). Further, the glycerol-plasticized films displayed ~2 to 4 times lower TS than the sorbitol plasticized films. Basiak et al. [87] compared the wheat, corn, and potato starches films for their mechanical properties. They concluded that wheat starch film is more deformable (EAB%) and less stiff (TS and Young modulus) than potato starch films. They added that the mechanical resistance of the films is more related to the thickness. The greater the thickness, the higher will be the TS. The mechanical characteristics of starch films strongly depend on the water content due to its hydrophilic nature; hence the lower amylose content significantly favors higher TS and Young modulus of films made from potato, wheat, and corn starches, respectively. Jha et al. [97,98] reported amylose–amylopectin ratios of 28:72 in corn starch-based films were found to have shown higher tensile strength, lower WVP, higher *T*_g_, and higher thermal stability, compared to high amylose corn starch, 70:30; wheat starch, 25:75; or potato starch, 20:80. It seems that the ratio amylose:amylopectin regulates the orientation of molecular structure in the starch-based films.

Song et al. [99] evaluated the mechanical characteristics of corn and wheat (6:4) starch films and reported TS and EAB of 15.50 MPa and 30%. They noted that essential oil in starch films decreased TS and EAB by 28.41% and 19.82% compared to the control film. A decrease in TS could be due to essential oils, which reduce the TS by developing a heterogeneous film structure featuring discontinuities. In addition, Basiak et al. [87] also observed a decrease in TS and Young modulus; however, EAB was decreased while adding rapeseed oil in the lamination technique. The incorporation of oil induced a two times reduction in the TS, as observed by several authors for both polysaccharide and protein film containing oils as emulsions [100]. Native and octenyl succinate modified sweet potato starch was used by Li et al. [101] for film formation. They reported that octenyl succinate modified sweet potato starch films exhibited higher EAB but lower TS values, confirming the potential of starch modification to improve the stretchability of films.

Incorporating nanoparticles as reinforcers and fillers into food packaging materials is reported to exhibit improved mechanical properties compared to the pure starch films. Nanoclay, nanocellulose, and nano silicon dioxide are examples of additive that is widely available, cost-effective, and biodegradable and has been shown to improve the properties of various polymer materials. Sadegh-Hassani and Nafchi [102] formulated potato starch films with a mixture of sorbitol/glycerol by adding nanoclay. Results showed that by increasing the concentration of nanoclay, the mechanical properties of films were improved. TS was increased from 7.33 to 9.82 MPa, and EAB decreased from 68.0 to 44.0%, and these nanocomposites have a high potential for food packaging purposes. The addition of nanoclays (e.g., organo MMT, halloysite, and sepiolite) is characterized by the adhesion between the polymer matrix and the unmodified nanoclay, resulting in improvement in film properties, such as mechanical strength of freestanding copolymer nanocomposite films. Therefore, starch weakness can be overcome by the addition of nanoclays. Furthermore, the application of nanoclays can be extended as antimicrobial agents (e.g., against *Staphylococcus aureus* (*S. aureus*), *Escherichia coli* (*E. coli*), *Salmonella typhimurium*, *Listeria monocytogenes*), to control and to release active ingredients, as colorimetric indicator template for intelligent packaging, and biodegradability stimulator [103,104,105,106,107,108].

A biodegradable colorimetric indicator starch–clay nanocomposite film was developed to monitor milk spoilage and was attached to the milk bottle. The water solubility of the starch-based film was reduced by adding nanoclay and restrained a dye release phenomenon into the milk [109].

The incorporation of nanoparticles occupies the sites on starch that normally would be occupied by water [102]. Mechanical properties of the composite films have been reported to be highly dependent on the interfacial interaction between the matrix and fillers. Wu et al. [110] showed that nanoparticles, used as a filling agent, improve the wear performance and tensile strength of starch films. The nanoparticles are likely to bond with hydroxyl groups and other possible hydrogen or Van der Walls bonds of starch macromolecules strengthening molecular forces between nanoparticles and starch.

However, there is still a restriction in using nanocomposite films in food packaging due to legislation surrounding nanomaterials lack of respect for consumer and environmental safety.

**Table 1:** Barrier properties of the starch-based film.

Blends	-	Water Vapor Permeability	Oxygen Permeability	References
Corn	38 °C and 90% RH	3.86 g·mm^−2^·s^−1^·Pa^−1^	-	Wang et al. [111]
Corn	Zanthoxylum bungeanum essential oil (0.5–2%), 38 °C and 90% RH	3.01–3.67 g·mm^−2^·s^−1^·Pa^−1^	-	Wang et al. [111]
Uncomplexed maize starch	38 °C, 10 ± 0.82% RH	30.1 g·mm·m^−2^·h^−1^·kPa^−1^	206 cm^3^·μm·cm^−2^·d^−1^·kPa^−1^	Teklehaimanot et al. [85]
Zein	38 °C, 10 ± 0.82% RH	15.1 g·mm·m^−2^·h^−1^·kPa^−1^	569 cm^3^·μm·cm^−2^·d^−1^·kPa^−1^	Teklehaimanot et al. [85]
Maize starch and zein blend films	Uncomplexed maize starch (33–75%) + Maize starch complexed (25–33%) with stearic acid + Zein (0–50%), 38 °C, 10 ± 0.82% RH	9.6–15.5 g·mm·m^−2^·h^−1^·KPa^−1^	336–588 cm^3^·μm·cm^−2^·d^−1^·kPa^−1^	Teklehaimanot et al. [85]
Banana Starch	38 °C, 58% RH	0.19 g·mm·h^−1^·m^−2^·kPa^−1^	-	Silva et al. [112]
Faba bean starch film	25 °C, 75% RH	1.34 g·mPa^−1^·s^−1^·m^−2^	-	Sharma et al. [65]
Faba bean starch film	Crosslinkage at 1–5%, 25 °C, 75% RH	1.10–1.25 g·mPa^−1^·s^−1^·m^−2^	-	Sharma et al. [65]
Mungbean starch	Unplasticized, 23 °C, 100% RH		1.15 cc·µm/m^2^ day kPa	Rompothi et al. [78]
	Glycerol (20–30%), 23 °C, 100% RH		0.8102–1.1394 cc·µm/m^2^ day kPa	Rompothi et al. [78]
	Sorbitol (20–30%), 23 °C, 100% RH		0.4493–0.5002 cc·µm/m^2^ day kPa	Rompothi et al. [78]
Rapeseed laminated plasticized wheat starch film				
	25 °C, 33–0% RH	0.56 (10^−10^ g·m^−1^·s^−1^·Pa^−1^) (3% starch) 0.92 (10^−10^ g·m^−1^·s^−1^·Pa^−1^) (5% starch) 0.28 (10^−10^ g·m^−1^·s^−1^·Pa^−1^) (3% starch + oil) 0.57 (10^−10^ g·m^−1^·s^−1^·Pa^−1^) (5% starch + oil)		Basiak et al. [87]
	25 °C, 75–30% RH	4.55 (10^−10^ g·m^−1^·s^−1^·Pa^−1^) (3% starch) 8.77 (10^−10^ g·m^−1^·s^−1^·Pa^−1^) (5% starch) 0.25 (10^−10^ g·m^−1^·s^−1^·Pa^−1^) (3% starch + oil) 3.55 (10^−10^ g·m^−1^·s^−1^·Pa^−1^) (5% starch + oil)		Basiak et al. [87]
	25 °C, 100–30% RH	3.98 (10^−10^ g·m^−1^·s^−1^·Pa^−1^) (3% starch) 8.01 (10^−10^ g·m^−1^·s^−1^·Pa^−1^) (5% starch) 0.92 (10^−10^ g·m^−1^·s^−1^·Pa^−1^) (3% starch + oil) 3.40 (10^−10^ g·m^−1^·s^−1^·Pa^−1^) (5% starch + oil)		Basiak et al. [87]
	25 °C, 33% RH		7.55 (10^−14^ cm^−3^·m^−1^·s^−1^·Pa^−1^) (3% starch) 7.23 (10^−14^ cm^−3^·m^−1^·s^−1^·Pa^−1^) (5% starch) 0.14 (10^−14^cm^−3^·m^−1^·s^−1^·Pa^−1^) (3% starch + oil) 0.96 (10^−14^ cm^−3^·m^−1^·s^−1^·Pa^−1^) (5% starch + oil)	Basiak et al. [87]
	25 °C, 75% RH		6.63 (10^−14^ cm^−3^·m^−1^·s^−1^·Pa^−1^) (3% starch) 7.41 (10^−14^ cm^−3^·m^−1^·s^−1^·Pa^−1^) (5% starch) 1.00 (10^−14^ cm^−3^·m^−1^·s^−1^·Pa^−1^) (3% starch + oil) 1.12 (10^−14^ cm^−3^·m^−1^·s^−1^·Pa^−1^) (5% starch + oil)	Basiak et al. [87]

**Table 2:** Physical and mechanical properties of starch-based films.

Type of Starch Used	Solubility	Film Thickness (µm)	*Moisture Content* (%)	Tensile Strength (MPa)	Elongation at Break (%)	*Young Modulus* (MPa)	Reference
Maize starch	-	266	22.26	1.49	51	14.2	Żołek-Tryznowska and Kałuża [113]
Potato starch	-	332	9.74	3.05	70	14.5	Żołek-Tryznowska and Kałuża [113]
Oat starch	-	266	21.77	0.36	27	1.8	Żołek-Tryznowska and Kałuża [113]
Rice starch	-	145	18.72	1.80	49	9.6	Żołek-Tryznowska and Kałuża [113]
Topaca starch	-	136	17.22	0.78	137	0.8	Żołek-Tryznowska and Kałuża [113]
Corn and wheat	46.16–33.45	72.55–77.27	23.20–10.08	15.50	30.00	-	Song et al. [99]
Wheat	30.16	74.1	0.445	3.29	15.21	0.12	Basiak et al. [87]
Corn	44.76	112.2	0.367	3.72	19.13	0.10	Basiak et al. [87]
Potato	14.52	55.4	0.316	6.56	5.67	5.33	Basiak et al. [87]
Cassava starch, mungbean starch, Cassava + mungbean starch	-	-	-	2.86–20.64	10.84–21.37	-	Vu et al. [114]
Wheat starch	14.49–19.67	35.4–80.8	2.01–3.24	2.03–2.10	13.18–14.16	0.08–0.10	Basiak et al. [87]
Wheat starch laminated with oil	7.83–10.70	22.1–27.7	2.69–2.70	0.92–1.04	16.44–18.29	0.03	Basiak et al. [87]
Sweet potato starch	19.99	0.106	15.20	-	-	-	Li et al. [101]
OSA modified sweet potato starch	15.25–19.72	0.091–098	13.41–14.13	-	-	-	Li et al. [101]
Potato starch	35	-	-	7.33	68	188	Sadegh-Hassani, and Nafchi [102]
Potato starch + Nanoclay (1, 2, 3 and 5%)	23–30	-	-	8.09–9.82	44–61.5	297–376	Sadegh-Hassani, and Nafchi [102]
Potato starch	14.26–19.87	0.073–0.168	-	4.87–5.25	56.87–85.20	3.95–9.25	da Rosa Zavareze et al. [115]
Heat and moisture treated potato starch	18.89–19.89	0.102–0.124	-	6.07–9.12	38.80–84.90	7.35–24.91	da Rosa Zavareze et al. [115]
Oxidized potato starch	14.78–18.87	0.118–0.147	-	6.38–7.24	79.93–84.20	8.01–8.61	da Rosa Zavareze et al. [115]

### 3.3. Optical Properties

Convenient, transparent, and easy-to-use starch films without any foreign particles (insoluble) could be prepared using yam starch (plasticized) [116]. While preparing to coat eatable materials, optical parameters, especially opacity, are considered significant determinant factors [117,118]. The addition of calcium carbonate nanoparticles in film prepared using corn starch results in higher opacity comparable to pure film (native corn starch) [119]. A decrease in transmittance was observed upon the addition of talc powder in composite films (Cassava starch–kaolinite) [120,121,122] (Table 3). Shi et al. [123] demonstrated that nanoparticles with an approximately similar size helps them incorporate into starch films, resulting in higher opacity (low light transmission). Ordered zones in films (starch) lead to a reduction in absorbance and increment in the transparency of films [123]. More absorbance of light is usually recommended as a desirable feature in food packing material as it helps prevent light-mediated oxidation of lipids.

Although glycerol is required to achieve transparency in films (starch), it should be added in an optimized amount as the increase in glycerol concentration leads to less transparency. Geleta et al. [124] prepared films (onset starch) using glycerol (15–25%), and they observed a decline in transparency with the rise in glycerol concentration. The transparency value they observed with varying glycerol concentrations was 85% (15% glycerol), followed by 81.7% (20%) and 78.4% (25%), respectively. However, the transparency level may also vary with the starch type and their mother source. Limpisophon et al. [125] observed that gelatin (blue-colored shark skin) was used in combination with glycerol (25%) resulted in more transparency in films.

Further, drying temperature also affects the transparency of film up to certain specified limits. Prabhu et al. [126] supported the concept that botanical sources also affect film clarity as they found significantly different optical values of films (teff starch and cassava starch). Protein isolated from peanut meal could also be used to prepare films; however, an increase in concentration affects the transparency and thickness of films [127,128]. Cheftel et al. [129] observed that the yellowish coloration in films might be due to the interaction of proteins with aldehydes or Maillard reactions with final/intermediate complexes. Inclusion of whey protein in starch (corn) or blend (methylcellulose) decreases light transmission. However, for commercial purposes, colored compounds may be added in edible films to reduce excessive oxidation [130,131]. Homogenous and transparent films could be prepared using a suitable amount of pea starch along with guar-gum and glycerol. They recommend that, compared to other components, starch be used in a higher amount to achieve desirable features in the films [132,133]. Loss in film transparency level might be due to the high amount of guar-gum, which results in phase separation as amylopectin and amylose start their interaction to form a network of hydrocolloid structures. The information is viable for preparing films at the commercial level to pack and coat food material and improve the appearance of food products [134].

The addition of oil extracted from seeds (sunflower) in starch films decreases transparency [135]. This might be due to the altered light passing from the film [83]. Improvement in light transmittance was observed when chitosan was used along with tapioca starch. The transmittance value observed for native and chitosan-added formulations was 84.7 and 85.3%, respectively [136]. Their finding helps to achieve significant transparency in films prepared for industrial uses.

Further, metal oxides are often used to extend or improve the functional properties of biodegradable films. TiO_2_ has been used extensively in food and cosmetic applications to block light and give a white appearance in compliance with the recommended safe dosage. Oleyaei et al. [137] modified starch film characteristics by including a limited content of nano-TiO_2_. The authors reported that the presence of TiO_2_ in the film matrix caused a remarkable absorbance of UV light even at the low contents of nanoparticles.

**Table 3:** Optical parameters of starch-based films.

Films		Opacity (%/mm)	References
Teff-starch		up to 85% Transparency	Prabhu et al. [126]
Cassava-starch		up to 81% Transparency	
Enset Starch	G1	85.08% Transparency	Geleta et al. [124]
	G2	81.72% Transparency	
	G3	78.46% Transparency	
	G4	80.94% Transparency	
	G5	80.36% Transparency	
	G6	83.96% Transparency	
Mung Bean starch	GG/MBS	19.77% Transparency	Lee et al. [135]
	0.5% SSO/GG/MBS	17.86% Transparency	
	1% SSO/GG/MBS	16.66% Transparency	
	2% SSO/GG/MBS	14.70% Transparency	
Pea starch and guar gum	2–3 (g)	82.38–84.91% Transparency	Saberi et al. [132]
Tapioca starch	0%	84.78% Transparency	Shapi and Othman [136]
	20%	85.34% Transparency	
	40%	85.29% Transparency	
	60%	85.21% Transparency	
	80%	85.80% Transparency	
Corn Starch	CS + 0.02% Ca	1.2960.21	Sun et al. [127]
	CS + 0.04% Ca	1.4560.16	
	CS + 0.06% Ca	1.6260.15	
	CS + 0.1% Ca	1.9460.06	
	CS + 0.5% Ca	2.2360.16	
Pea starch and Peanut protein isolates blend	F100:0	2.16	Sun et al. [127]
	F80:20	3.21	
	F60:40	4.19	
	F50:50	4.94	
	F0:100	5.59	
Yam starch		85.0 and 111.2 Au nm	Mali et al. [116]

The 100 nm Nano-SiO_2_/potato starch films obtained by Zhang et al., showed resistance against UV light and restrained the deterioration of food caused by UV radiation due to nano-SiO_2_ causing low transmittance [138]. Potato starch/cellulose nanocomposite (isolated from pineapple leaf) films were found to have higher transparency and to be UV resistant [139].

### 3.4. Biodegradability

Biodegradability defines the ability of a material to decompose into simpler ones after interacting with biological elements [140]. For plastic based-packaging industries, biodegradability is a significant area of concern. The use of non-biodegradable polymers affects the environment and climate adversely. In this regard, researchers’ interest is increasing towards using eco-friendly packaging materials. Therefore, a polymer with improved biodegradation properties could be a better replacement to mitigate biodegradation problems. Biobased and biodegradable polymers have many applications, including pharmaceutical, biomedical, horticulture, agriculture, automotive, textiles, and packaging. The development and application of biodegradable starch-based materials have attracted increasing attention due to the well-recognized issues of oil shortage and the growing interest in easing the environmental burden due to extensive use of petrochemically-derived polymers. Twelve Andean crops (tubers, legumes, roots, and fruits) were used for manufacturing starch films [141]. The authors used the cellulose film as the control one. They reported that after 31 days, the highest weight loss was observed for cassava starch-based films (99.35%); however, the lowest percentage value was reported for gold potato starch-based films (90.03%). In addition, the cellulose control film decreased 30% in weight in the same period. The higher weight loss in starch films could easily disrupt glycosidic alpha linkages than glycosidic beta linkages in cellulose. To evaluate the biodegradability of acid-soluble chitosan–starch-based film blended in lactic, formic, and acetic acid, Rachmawati et al. [142] used the soil burial test and used HDPE plastic as control film. All the films degraded naturally in a slightly similar period, ranging from 72 to 87 days. However, the higher plasticizer concentration reduced the biodegradability period from 81 to 72 days and 101 to 74 days for films diluted in acetic acid and formic acid, respectively. The blending of native or plasticized starch into PVA increased the biodegradability of polymer blends at a low starch level of 5 wt% [143].

Dominici et al. [144] investigated the composability of thermoplastic starch and poly(butylene cyclohexane dicarboxylate) containing 25% adipic acid. The authors reported that all components of the formulation of the film contributed to the achievement of novel eco-friendly material with fully bio-based character, high flexibility, good moisture resistance, with fast degradability in compost. Ojogbo et al. [145] investigate the reinforcement of corn starch ester films with sustainable nanofillers, including cellulose nanocrystals and nanoclay (montmorillonite organoclay modified with quaternary ammonium salts). They reported that incorporating fillers into the polymer improved the overall composability revealed by increased polymer weight loss with increased filler concentration.

## 4. Applications

Starch-based biodegradable materials need to meet specific mechanical, barrier, antibacterial, and antioxidant characteristics for food packaging materials. Improved food quality, shelf life, and safety are the essential attributes of food packaging materials. Many attempts have been made to improve the barrier (water and oxygen permeability) by blending with other polymers or adding antioxidants/antimicrobial agents in film-forming materials to extend the shelf life of foods. The films can suppress respiration and delay oxidation [146,147,148,149].

Starch-based films have been applied to packaging various foods, such as fruits and vegetables, bakery goods, and meat, indicating good prospects for commercial utilization (Table 4).

### 4.1. Active Packaging

Coating of strawberries with 3% cassava starch (amylose content 17–19%) and potassium sorbate (0.05%) reduced the respiration rate, improved the water vapor permeability, and provided good sensorial attributes to strawberries [150]. During the storage duration, food material packed using edible films may provide a platform for microbial spoilage. Oil with antimicrobial potential is used as additives to improve the shelf life and avoid pathogenic attacks on edible film-based packing. The addition of essential oils (exerting antimicrobial and antibacterial properties) in films significantly reduces the growth of microbial consortia on the surface of food. Adding essential oils into starch coatings is another approach to control pathogens and extend the shelf life of minimally processed fruits and vegetables. A 2–3% cassava starch coating containing carvacrol sufficiently inhibited pathogens in minimally processed pumpkins [151], and papaya [152] prevented weight loss and delay in fruit ripening.

Apart from fruits and vegetables, starch-based edible coatings are also applied to nuts and bakery products. Due to the better flexibility of rice starch-based films than wheat and cornstarch-based films, rice starch was suggested as a suitable coating for walnuts [153,154]. Rice starch plasticized with 2% glycerol coated a uniform layer and served as a barrier that decreases oxygen, moisture, and heat on walnuts. Further, the starch-based coating reduces storage space by removing the husk and shell of walnuts, resulting in fewer space requirements. Furthermore, chitosan and red palm oil incorporation provided a smoother layer because of compactness and homogeneity in the matrix [153]. Modified corn starch (by ascorbic acid) and tomato powder have the potential to extend the shelf life of bread made from frozen dough [154]. The authors observed an improved volume and texture of coated bread than the uncoated one. An increase in bread volume could be because starch and a tomato powder coating maintain certain moisture in the dough, resulting in improved gluten network and starch interaction. The bread coated with the edible coating displayed a greater interaction between the gluten network and the starch exhibit. This justifies the higher specific volume obtained by this bread. This behavior can be attributed to the coating promoting greater moisture to the dough and corroborating the fermentative process as previously described, which provides a more homogeneous gluten–starch interaction. Moreover, the nutritional profile of starch and tomato and the availability of more glucose molecules due to acid hydrolysis possibly promoted yeast growth [154]. Zhang et al. [138] added nano-TiO_2_ particles to the potato starch to formulate composite film and observed better barrier, mechanical, and antibacterial properties, which could be used as packaging material for white mushrooms preservation.

Incorporating antimicrobial agents into starch-based materials can release a controlled amount of active ingredients and reduce their interaction with other food compounds [155]. Starch-based intelligent food packaging has been prepared using grape seed extract to inhibit *Brochothrix thermosphacta* [156] and pomegranate peel particles to reduce the growth of *Salmonella* and *Staphylococcus aureus* [157]. In addition, Xu et al. [158] added Viognier (a phenolic compound) present in grape pomace extracts in corn starch film reinforced with cellulose nanocrystals. The authors concluded that films inhibited Listeria monocytogenes and Staphylococcus aureus growth. To evaluate the antimicrobial potential of films, sliced chicken deli meat pre-inoculated with Listeria monocytogenes was kept on the films, and results found that films enriched with grape pomace extract inhibited L. monocytogenes growth when stored at 4 °C for ten days [158]. Requena et al. [159] prepared thermo-processed polyester films using amorphous PLA and poly(3-hydroxybutyrate-co-3-hydroxy valerate), and further polyester monolayer films were combined with cassava starch to prepare bilayer films. The bilayer films could inhibit the growth of *Listeria innocua* and *Escherichia coli* depending on the internal diffusion of carvacrol through the bilayer. Garrido-Miranda et al. [160] also incorporated the eugenol into nanocomposites made from poly(3-hydroxybutyrate), corn starch, and organically modified montmorillonite to inhibit the growth of Botrytis cinerea. For inhibiting fungal growth, Noorbakhsh-Soltani et al. [161] blended gelatin and starch with nanocellulose and chitosan to prepare nanocomposites, and results found that composite films inhibited the fungal growth on pomegranate seeds stored at 25 °C for 14 days. Similar findings were observed by Castillo et al. [162] for yeast growth on bread, strawberry, and cheese when chitosan and thermoplastic corn starch was used as a packaging sachet.

To evaluate starch-based films’ antioxidant potential, Piñeros-Hernandez et al. [163] incorporated rosemary nanoparticles in plasticized cassava starch films. They reported that rosemary nanoparticles enhanced the antioxidant potential of films, being a good source of phenolics. These active films could be used for the controlled release of fatty food simulants. Farrag et al. [164] used pea and corn starches to encapsulate quercetin and formed starch–quercetin microparticles. These microparticles were used to prepare films that exhibited more heat stability than the controls. The pea starch films containing pea starch–quercetin microparticles displayed a higher loading percentage and higher radical scavenging activity than corn starch films with corn starch–quercetin microparticles.

A bio-hybrid material containing porous starch, halloysite nanotubes, and the antioxidant fucoxanthin was developed by Oliyaei et al. [165]. Fucoxanthin is heat and light sensitive and gradually releases with time. Incorporating fucoxanthin in halloysite nanotubes and starch improved its potential during four weeks of storage when exposed to sunlight. The authors concluded that results provide a base to develop bio-composite films as antioxidant releasing systems. Tongdeesoontorn et al. [166] prepared cassava starch/gelatin active food packaging integrated with quercetin and tertiary butylhydroquinone (TBHQ) and reported that these composite films could be utilized as active packaging that delays oxidation in foods. Further, García et al. [91] developed corn starch-based edible films as low-price and sustainable food packaging systems to prevent the oxidative deterioration of packaged foodstuff. The authors incorporated olive extracts into the corn starch/glycerol matrix and showed the antioxidant and antimicrobial potential.

### 4.2. Intelligent Packaging

Bio-based smart packaging is a potential option, where sustainability and real-time monitoring of food quality are combined, assuring health safety and providing economic and environmental benefits. Different intelligent starch bases for packaging applications have been developed in the recent decade. Already some examples have been highlighted in this review, and a couple of other examples will be presented. An intelligent function of packaging is related to communicating, detecting, recording, sensing, tracing, aiming to provide information, facilitating the decision to extend shelf life, enhance safety, improve quality, and warn about possible problems [167].

Different components of plant extracts or natural dyes, such as chlorophyll and carotenoids, possess the property of giving yellow-green pigmentation and undergo color changes when exposed to fluctuating pH conditions. The changes in pH of a food product indicate changes in state and quality; therefore, pH is a valuable and essential indicator. In general, the natural bioactive compounds used to obtain intelligent films for packaging applications possess antimicrobial and antioxidant activities; therefore, intelligent packaging is, in most cases, active packaging at the same time, but few studies are evaluating both functions. If both active and intelligent functions are considered, then the packaging is presented as smart biodegradable packaging [167,168].

Starch-based intelligent food packaging can be used as a visual indicator of freshness to monitor the user to monitor shrimp freshness. Choi et al. [169] prepared a new colorimetric pH indicator film using agar, potato starch, and natural dyes extracted from purple sweet potato. The pH indicator films showed pH changes and spoilage points of pork samples, changing from red to green. The authors concluded that these films could be used as a diagnostic tool to detect food spoilage.

The purple sweet potato anthocyanins have been incorporated into carboxymethyl-cellulose (CMC)/starch as an indicator to monitor the real-time freshness of raw fish (grass carp) stored at 20 °C, shifting color from red to blue and green when exposed to different pH or ammonia [170].

In another study, purple sweet potato was added to starch/PVA films for simultaneous colorimetric indication and antimicrobial activity for smart food packaging applications [37].

Medina-Jaramillo et al. [167] prepared active and intelligent packaging by incorporating green tea and basil in cassava starch and glycerol by casting method. Chlorophyll and carotenoids in green tea and basil extracts change the color when exposed to different pH, resulting in materials to be used as food quality indicators.

Further, Zhang et al. [171] extracted anthocyanin from cabbage and sweet potato and incorporated it into starch and polyvinyl alcohol films to indicate shrimp freshness. They prepared the pH-sensitive films that could be used as an indicator to monitor the shrimp freshness and exhibited effective color change during shrimp spoilage. Another intelligent packaging to monitor the freshness of shrimp has been developed using a starch/polyvinyl alcohol film base incorporating betacyanins from different plant sources, such as red pitaya flesh extract (RPFE), prickly pear fruit extract (PPFE), red beetroot extract (RBRE), globe amaranth flower extract (GAFE), and red amaranth leaf extract (RALE). The pH changes caused a color change from pink to yellow [172,173].

Red cabbage (*Brassica oleraceae*) anthocyanins added to chitosan/corn starch-based biopolymer mixture have been implemented as a visual indicator of fish fillet deterioration [174]. Cassava starch has been studied for different applications for pH indicator intelligent food packaging films combined with anthocyanins from Lycium ruthenicum Mur, blueberry residue and pomace, or blueberry pomace [175,176,177,178] Anthocyanins from grape skin incorporated into a cassava starch sheet produced by the extrusion process have been used for beef and fish products acting as a pH-sensor, showing color changes when the pH environment changed [175].

Starch/PVA composite packaging film with Roselle anthocyanins has been developed to monitor raw fish freshness (silver carp). An intelligent pH sensing wraps for food packaging applications has been obtained by incorporating Jamun (*Syzygium cumini*) anthocyanins into starch/PVA film composite with zinc-oxide nanoparticles [179]. Starch and gelatin films containing anthocyanins from red radish changed their color in a pH ranging from 2 to 12, from red to grey-purple, as a response to volatile nitrogenous compounds produced by spoilage prawn and poultry meat.

**Table 4:** Highlights of applications of starch-based films on food products.

Starch	Additive/s	Product	Application	Findings	References
Corn starch	Anthocyanin and polyvinyl alcohol	-	-	Used as an indicator to check the freshness of seafood	Zhang et al. [171]
Hydroxypropyl high-amylose starch	Pomegranate peel	-	Film	↓ *Salmonella* and *Staphylococcus aureus*	Ali et al. [157]
Potato starch	Nano-SiO_2_	White mushrooms	Films	Preserve white mushrooms	Zhang et al. [138]
Starch	Cellulose nanocrystals and chitosan	Pomegranate seeds	-	↓ fungal growth	Noorbakhsh-Soltani et al. [161]
Corn starch	Eugenol, poly(3-hydroxybutyrate) and montmorillonite	-	Film	↓ *Botrytis cinerea*	Garrido-Miranda et al. [160]
Cassava starch	Carvacrol and PLA and poly(3-hydroxybutyrate-co-3 hydroxy valerate)	-	film	↓ *Listeria innocua* ↓ *Escherichia coli.*	Requena et al. [159]
Modified corn starch	Tomato powder	Bread from frozen dough	Edible coating	↑ Specific volume ↑ Texture	Galvão et al. [154]
Thermoplastic corn starch	Chitosan	Bread, strawberry, and cheese	Sachet	↓ Yeast growth	Castillo et al. [162]
Cassava starch	Rosemary nanoparticles	-	Active films	↑Antioxidant activity	Piñeros-Hernandez et al. [163]
Rice starch	Chitosan + red palm oil	Walnut	Coating	↓Oxygen, moisture, and heat effect	Aghazadeh et al. [153]
Corn starch	Grape pomace extracts and cellulose nanocrystals	-	Film	↓ *Listeria monocytogenes* ↓ *Staphylococcus aureus*	Xu et al. [158]
Cassava starch	Carvacrol	Pumpkins	Edible coating	↑ Shelf life ↓ Pathogens	Santos et al. [151]
Cassava starch	-	Papaya	Edible coating	↓ Fruit ripening ↓ *Anthracnose*	De Oliveira et al. [152]
Cassava starch	Potassium sorbate	Strawberry	Edible coating	↓ Respiration rate ↑ Water vapor permeability	Garcia et al. [150]
Pea starch	Grape seed extracts	Boneless pork loin	Edible coating	↓ *Brochothrix thermosphacta* growth	Corrales et al. [156]

## 5. Conclusions and Future Direction

Starch has received considerable attention for biodegradable film formulation due to its biodegradability, edible, low cost, ease of use, and thermoprocessable nature. Starch-based films and coatings have been considered an alternative to conventional packaging to improve food quality and safety. Additionally, starch-based films are used as carriers of functional ingredients to prepare active, antioxidant, and intelligent packaging by incorporating antimicrobial, antibrowning, and nutraceutical agents to improve shelf-life and quality.

However, developing new technologies to improve the delivery properties of films and coatings still requires future research. Most studies on food applications have been conducted at a laboratory scale; thus, research on cost reduction and larger-scale production and stability and safety is necessary to promote the feasibility of commercialized biodegradable packaged or coated products. Further studies should optimize film formulation and processing conditions to enhance humidity susceptibility and film properties for specific applications.

## Data Availability

The data presented in this study are available on request from the corresponding author.
